# Brief motivational intervention for adolescents treated in emergency departments for acute alcohol intoxication - a randomized-controlled trial

**DOI:** 10.1186/1940-0640-10-S2-O14

**Published:** 2015-09-24

**Authors:** Silke Diestelkamp, Nicolas Arnaud, Lutz Wartberg, Anne Daubmann, Rainer Thomasius

**Affiliations:** 1 German Center for Addiction Research in Childhood and Adolescence, University Medical Center Hamburg-Eppendorf, Hamburg, Germany; 2 Department of Medical Biometry and Epidemiology, University Medical Center Hamburg-Eppendorf, Hamburg, Germany

## Background

Rising numbers of adolescents receiving emergency medical treatment due to acute alcohol intoxication have been a major public health concern in a range of European countries in recent years. Brief interventions addressing this target population have been introduced in a number of emergency departments with the aim to reduce alcohol-related harm. The “HaLT-Hamburg” trial evaluated effectiveness of a manualized brief motivational intervention addressing under 18 year-olds following alcohol intoxication in this setting. To our knowledge, we are the first to evaluate a brief intervention for the special target group of adolescents with acute alcohol intoxication in a randomized-controlled design.

## Material and methods

The trial design is a parallel two-arm cluster randomized-controlled trial with follow-up assessment after 3 and 6 months[1]. Children and adolescents with the diagnosis acute alcohol intoxication (ICD-10 F10.0) were recruited in 6 urban emergency departments over a period of 30 months. Intervention condition was a manual-based brief motivational intervention with a telephone booster after 6 weeks and a manual-guided intervention for caregivers. Control condition was treatment as usual (information leaflet). Primary outcomes were reduction in binge drinking episodes, quantity of alcohol use on a typical drinking day and alcohol-related problems (RAPI). Linear mixed models adjusted for baseline differences were conducted according to intention-to-treat (ITT) and completers (per-protocol) principles to examine intervention effects.

## Results

N = 316 adolescents with a mean age of 15.8 years (SD = 1.16) were included in the study. Both conditions resulted in reduced binge drinking episodes, quantity of alcohol use on a typical drinking day and alcohol-related problems at 3 month follow-up and stayed at a low rate at 6 month follow-up.

## Conclusions

Intervention effects and subgroup analyses will be presented and clinical implications for the delivery of brief interventions to adolescents with acute alcohol intoxication will be discussed.

## Trial registration

Current Controlled Trials ISRCTN31234060.
